# Supplementary material for: The association between 25(OH)D levels, frailty status and obesity indices in older adults
Source: PLoS One. 2018 Aug 28;13(8):e0198650. doi: 10.1371/journal.pone.0198650 (PMC6112621; doi:10.1371/journal.pone.0198650)
Supplement: S1 Table — (DOCX) [file pone.0198650.s001.docx]

**S1 Table.** **Comparison of the included and excluded individuals^†^.**

|  | **N (%)** | |  |
| --- | --- | --- | --- |
|  | **Included**  **1447 (96.5)** | **Excluded**  **53 (3.5)** | ***p*-value** |
| **Sex** |  |  |  |
| Women | 837 (57.8) | 35 (66.0) | 0.235^a^ |
| Men | 610 (42.2) | 18 (34.0) |  |
| **Age** |  |  |  |
| 65-75 | 826 (57.1) | 27 (50.9) | 0.375^a^ |
| >75 | 621 (42.9) | 26 (49.1) |  |
| **Education level** |  |  |  |
| Without schooling | 205 (14.2) | 7 (13.2) | 0.886^b^ |
| 1-4 years | 992 (68.6) | 39 (73.6) |  |
| 5-12 years | 183 (12.6) | 6 (11.3) |  |
| >12 years | 67 (4.6) | 1 (1.9) |  |
| **Smoking status** |  |  |  |
| Non-smoker | 1381 (95.4) | 51 (96.2) | 1.000^b^ |
| Smoker | 66 (4.6) | 2 (3.8) |  |
| **Alcohol consumption** |  |  |  |
| None | 705 (48.7) | 34 (66.7) | 0.001^a^ |
| Moderate (W: ≤1/day; M: ≤2/day) | 597 (41.3) | 8 (15.7) |  |
| Excessive (W: >1/day; M: >2/day) | 145 (10.0) | 9 (17.6) |  |
| **Cognitive function (MMSE)** |  |  |  |
| Normal | 1355 (93.6) | 46 (86.8) | 0.049^a^ |
| Impaired | 92 (6.4) | 7 (13.2) |  |
| **Vitamin D (IOM)** |  |  |  |
| Adequacy | 449 (31.0) | 16 (30.2) | 0.908^a^ |
| Inadequacy | 424 (29.3) | 17 (32.1) |  |
| Deficiency | 574 (39.7) | 20 (37.7) |  |
| **Skin phenotype** |  |  |  |
| Red-haired with freckles or fair-haired people | 302 (20.9) | 3 (6.0) | 0.002^b^ |
| Dark-haired or Latin people | 1071 (74.0) | 40 (80.0) |  |
| Arab, Asian or Black people | 74 (5.1) | 7 (14.0) |  |
| **BMI categories** |  |  |  |
| Normal weight/ Underweight | 239 (16.5) | 13 (26.5) | 0.183^a^ |
| Pre-obesity | 642 (44.4) | 19 (38.8) |  |
| Obesity | 566 (39.1) | 17 (34.7) |  |
| **Waist circumference** |  |  |  |
| Women ≤80 cm; Men ≤94 cm | 179 (12.4) | 10 (18.9) | 0.053^b^ |
| Women: 81-88 cm; Men: 95-102 cm | 302 (20.9) | 11 (20.8) |  |
| Women >88 cm; Men >102 cm | 956 (66.1) | 30 (56.6) |  |
| Not evaluated | 10 (0.7) | 2 (3.8) |  |
| **BRI, median (IQR)** | 6.1 (2.4) | 6.1 (3.2) | 0.412^c^ |
| **ABSI (m^11/6^·kg^-2/3^), mean (SD)** | 0.084 (0.006) | 0.084 (0.006) | 0.888^d^ |
| **Season of blood collection** |  |  |  |
| Spring/Summer | 702 (48.5) | 29 (56.9) | 0.241^a^ |
| Autumn/Winter | 745 (51.5) | 22 (43.1) |  |
| **Vitamin D supplementation** |  |  |  |
| No use | 1218 (84.2) | 44 (83.0) | 0.873^b^ |
| Use of vitamin D supplements | 77 (5.3) | 3 (5.7) |  |
| Unknown use or composition | 152 (10.5) | 6 (11.3) |  |

W: Women; M: Men; MMSE: Mini Mental State Examination; BMI: Body mass index; WC: Waist circumference; BRI: Body roundness index; ABSI: Body shape index.

^†^Column percentages may not add to 100% due to rounding. Missing data in the excluded individuals: Body mass index (n=4); Alcohol consumption (n=2); Skin phenotype (n=3); Season of blood collection (n=2).

^a^ Qui-square test; ^b^ Fisher’s exact test; ^c^ Mann-Whitney test; ^d^ T-test
