# Supplementary material for: The association between 25(OH)D levels, frailty status and obesity indices in older adults
Source: PLoS One. 2018 Aug 28;13(8):e0198650. doi: 10.1371/journal.pone.0198650 (PMC6112621; doi:10.1371/journal.pone.0198650)
Supplement: S2 Table — (DOCX) [file pone.0198650.s002.docx]

**S2 Table.** **Characteristics of the 1447 study participants by obesity and frailty status.**

|  | **N (%)** | | | | |
| --- | --- | --- | --- | --- | --- |
|  | **Frailty(-) & Obesity(-)**  **723 (50.0)** | **Frailty(-) & Obesity(+)**  **414 (28.6)** | **Frailty(+) & Obesity(-)**  **158 (10.9)** | **Frailty(+) & Obesity(+)**  **152 (10.5)** | ***p*-value** |
| **Sex** |  |  |  |  |  |
| Women | 359 (49.7) | 263 (63.5) | 102 (64.6) | 113 (74.3) | <0.001^a^ |
| Men | 364 (50.3) | 151 (36.5) | 56 (35.4) | 39 (25.7) |  |
| **Age (years)** |  |  |  |  |  |
| 65-75 | 461 (63.8) | 269 (65.0) | 46 (29.1) | 50 (32.9) | <0.001^a^ |
| >75 | 262 (36.2) | 145 (35.0) | 112 (70.9) | 102 (67.1) |  |
| **Education level** |  |  |  |  |  |
| Without schooling | 75 (10.4) | 53 (12.8) | 36 (22.8) | 41 (27.0) | <0.001^a,e^ |
| 1-4 years | 481 (66.5) | 300 (72.5) | 107 (67.7) | 104 (68.4) |  |
| 5-12 years | 117 (16.2) | 48 (11.6) | 14 (8.9) | 4 (2.6) |  |
| >12 years | 50 (6.9) | 13 (3.1) | 1 (0.6) | 3 (2.0) |  |
| **Smoking status** |  |  |  |  |  |
| Non-smoker | 681 (94.2) | 403 (97.3) | 146 (92.4) | 151 (99.3) | 0.001^b^ |
| Smoker | 42 (5.8) | 11 (2.7) | 12 (7.6) | 1 (0.7) |  |
| **Alcohol consumption** |  |  |  |  |  |
| None | 308 (42.6) | 191 (46.1) | 114 (72.2) | 92 (60.5) | <0.001^a^ |
| Moderate (W: ≤1/day; M: ≤2/day) | 338 (46.7) | 173 (41.8) | 35 (22.2) | 51 (33.6) |  |
| Excessive (W: >1/day; M: >2/day) | 77 (10.7) | 50 (12.1) | 9 (5.7) | 9 (5.9) |  |
| **Cognitive function (MMSE)** |  |  |  |  |  |
| Not impaired | 684 (94.6) | 400 (96.6) | 137 (86.7) | 134 (88.2) | <0.001^a^ |
| Impaired | 39 (5.4) | 14 (3.4) | 21 (13.3) | 18 (11.8) |  |
| **WC (cm), median (IQR)^†^** | 94.5 (12.3) | 108.4 (12.2) | 93.0 (10.8) | 109.3 (12.5) | <0.001^c^ |
| **WC ^†^** |  |  |  |  |  |
| W: ≤80 cm; M: ≤94 cm | 150 (20.7) | 0 (0.0) | 29 (19.0) | 0 (0.0) | <0.001^b,f^ |
| W: 81-88 cm; M: 95-102 cm | 245 (33.9) | 15 (3.6) | 41 (26.8) | 1 (0.7) |  |
| W: >88 cm; M: >102 cm | 328 (45.4) | 399 (96.4) | 83 (54.2) | 146 (99.3) |  |
| **BRI, median (IQR)^†^** | 5.4 (1.5) | 7.6 (1.8) | 5.6 (1.5) | 8.1 (1.8) | <0.001^c^ |
| **ABSI (m^11/6^·kg^-2/3^), mean (SD)^†^** | 0.084 (0.006) | 0.083 (0.005) | 0.085 (0.006) | 0.084 (0.005) | 0.002^d^ |
| **25(OH)D (ng/mL), median (IQR)** | 17.1 (14.9) | 13.7 (13.1) | 10.1 (11.2) | 9.2 (10.9) | <0.001^c^ |
| **Vitamin D (IOM)** |  |  |  |  |  |
| Adequacy | 275 (38.0) | 118 (28.5) | 28 (17.7) | 28 (18.4) | <0.001^a^ |
| Inadequacy | 230 (31.8) | 127 (30.7) | 39 (24.7) | 28 (18.4) |  |
| Deficiency | 218 (30.2) | 169 (40.8) | 91 (57.6) | 96 (63.2) |  |
| **25(OH)D Quartiles** |  |  |  |  |  |
| Q1 (3.0-8.7 ng/mL) | 130 (18.0) | 95 (22.9) | 67 (42.4) | 68 (44.7) | <0.001^a^ |
| Q2 (8.8-14.3 ng/mL) | 160 (22.1) | 122 (29.5) | 42 (26.6) | 40 (26.3) |  |
| Q3 (14.4-22.9 ng/mL) | 210 (29.0) | 106 (25.6) | 23 (14.6) | 22 (14.5) |  |
| Q4 (23.0-178.1 ng/mL) | 223 (30.8) | 91 (22.0) | 26 (16.5) | 22 (14.5) |  |
| **Skin phenotype** |  |  |  |  |  |
| Red-haired with freckles or fair‑haired people | 149 (20.6) | 75 (18.1) | 34 (21.5) | 44 (28.9) | 0.007^a^ |
| Dark-haired or Latin people | 542 (75.0) | 318 (76.8) | 108 (68.4) | 103 (67.8) |  |
| Arab, Asian or Black people | 32 (4.4) | 21 (5.1) | 16 (10.1) | 5 (3.3) |  |
| **Season of blood collection** |  |  |  |  |  |
| Spring/Summer | 401 (55.5) | 202 (48.8) | 50 (31.6) | 49 (32.2) | <0.001^a^ |
| Autumn/Winter | 322 (44.5) | 212 (51.2) | 108 (68.4) | 103 (67.8) |  |
| **Vitamin D supplementation** |  |  |  |  |  |
| No use | 624 (86.3) | 362 (87.4) | 110 (69.6) | 122 (80.3) | <0.001^a^ |
| Use of vitamin D supplements | 47 (6.5) | 20 (4.8) | 6 (3.8) | 4 (2.6) |  |
| Unknown use or composition | 52 (7.2) | 32 (7.7) | 42 (26.6) | 26 (17.1) |  |

W: Women; M: Men; MMSE: Mini Mental State Examination; WC: Waist circumference; BRI: Body roundness index; ABSI: Body shape index; SD: Standard Deviation; IQR: Interquartile range; IOM: Institute of Medicine. Column percentages may not add to 100% due to rounding.

^a^ Chi-square test; ^b^ Fisher’s exact test; ^c^ Kruskal-Wallis test; ^d^ ANOVA test; ^e^ *p*-value obtained with the last two categories compressed; ^f^ *p*‑value obtained considering WC as a dichotomous variable.

**^†^**Missing data in 10 individuals (0.7%).
