# Supplementary material for: The association between 25(OH)D levels, frailty status and obesity indices in older adults
Source: PLoS One. 2018 Aug 28;13(8):e0198650. doi: 10.1371/journal.pone.0198650 (PMC6112621; doi:10.1371/journal.pone.0198650)
Supplement: S3 Table — (DOCX) [file pone.0198650.s003.docx]

**S3 Table. Correlation between body mass index, waist circumference, body roundness index and body shape index.**

|  | **BMI** | **WC** | **BRI** | **ABSI** |
| --- | --- | --- | --- | --- |
| **BMI** | 1 |  |  |  |
| **WC** | 0.748* | 1 |  |  |
| **BRI** | 0.824* | 0.859* | 1 |  |
| **ABSI** | -0.121* | 0.476* | 0.358* | 1 |

BMI: Body mass index; WC: Waist circumference; BRI: Body roundness index; ABSI: Body shape index

**p*<0.001 obtained by Spearman correlation
